# Till debt does us apart: Cross-country evidence on the relationship between microfinance prevalence and social distrust

**DOI:** 10.1371/journal.pone.0282072

**Published:** 2023-03-08

**Authors:** Syed Muhammad Usman Masood, Rasim Özcan, Asad ul Islam Khan

**Affiliations:** Department of Economics, Ibn Haldun University, Başakşehir, Istanbul, Turkey; University of Agriculture Faisalabad, PAKISTAN

## Abstract

Economic interventions have social consequences. In this paper, we explore one such relationship, between microfinance intensity and social distrust levels reported by the low-income people. We find a significant association between microfinance intensity in a country and distrust among the poor as well as ultra-poor in cross-section using World Values Survey & European Values Survey (WVS-EVS) Wave 7 (2017–2022). We supplement these findings using empirical Bayes on a panel extending back from 7th to the 4th WVS wave (1999–2004). To deal with potential endogeneity, we run 2SLS as well as weak instruments-robust conditional instrumental variable tests and find evidence showing microfinance prevalence intensity affects distrust levels among the poor and ultra-poor households. We find no association between microfinance and distrust levels in the rich in any of the tests, potentially because the rich are not exposed to microfinance.

## 1. Introduction

Thanks to group-lending, the emblematic models of microfinance—Village Banking in Latin America and Grameen Bank in South Asia—offered mechanisms to tap social capital within communities in novel ways, making financial inclusion of the poor much easier. Joint liability, which the typical models of microfinance are based on [1], solved multiple problems from the lending institutions’ perspective. It provided a mechanism to avoid the problems of costly state-verification (utilising peer-monitoring), adverse selection (by relying on peer-screening) and moral hazard (with threat of financial and social exclusion), which had held back financial institutions from providing unsecured credit to the poor [2,3].

Yet the question of optimal leveraging of social capital for better wellbeings was elusive, and perhaps tedious to solve, and was left to credit markets [4,5]. On the one hand, microfinance institutions tweaked the parameters of the original models to make microfinance commercially viable [6]. On the other, the poor, diffuse, credit-constrained borrowers, welcomed the new profusion of loans, thanks to the immediate relief they offered, but without the requisite financial sophistication [7]. The findings on the effectiveness of microfinance in terms of financial wellbeing and income growth, or other related outcomes such as human development and women empowerment, are mixed [8–14].

A crucial element of the group-lending model, relatively understudied in economics, is the effect of the substitution of physical, by the social, collateral [15,16]. With any physical capital to pledge absent, a borrower in the group-lending model effectively pledges his/her social capital to obtain a loan. This means that a social cost is programmed into the model, which takes effect in case of delinquency or default. Crucially, this is different from the individual, economic cost associated with a default [17] in conventional, secured credit, where only the physical collateral is at stake.

This study focuses on one such social cost of microfinance, namely, social distrust (henceforth, distrust). Distrust may be a direct consequence of within-group default by a struggling member. With repayments required as frequently as every week, a borrower can easily fall behind the schedule and default. This triggers the peer pressure mechanism, which can materialise in the form of intense coercion and social sanctions by other members. Thus, the incentive structure of a typical joint-liability micro-loan pushes a credit-constrained borrower to convert a social relationship into an economic arrangement, which, when it derails, fractures the trust levels of borrowers, potentially affecting possibility of future cooperation [18].

Specifically, we seek to answer the question, “is microfinance prevalence (intensity) associated with social distrust among the low-income strata in a country?” and if there is evidence of microfinance causing distrust. Using data from the most recent, 7th wave/round (2017–2022) of the World Values Survey and European Values Survey integrated dataset (WVS-EVS) [19] for cross-sectional ordinary least squares (OLS), and the 4th to the 7th waves of the World Values Survey (WVS) for empirical Bayes panel analysis, we find the relationship between microfinance prevalence and distrust to be statistically significant for the poor as well as the ultra-poor (jointly, referred to as ‘low-income’). The relationship is not statistically significant for the rich, which is expected, as microfinance is targeted on only low-income households. To account for possible endogeneity issues, we use a novel instrument, i.e. percentage yield on microfinance loans adjusted for stability of yield (yield/standard deviation). The results for two-staged least squares (2SLS), as well as weak instruments-robust, coverage-corrected instrumental variable tests confirm the findings of the OLS and empirical Bayes.

The question explored in this study falls in the realm of an emerging strand in economics research which focuses on the social effects of economic interventions. Heß, Jaimovich, and Schündeln [20] study the effects of a community-driven development programme in Gambia in randomly selected treatment villages and find a significant reduction in informal economic interactions. They caution that development projects “can have unintended consequences for the economic and social networks of villages” [20]. Similarly, Comola and Prina [21] demonstrate that networks "may rewire" as a result of changes in economic environment, including financial interventions, and thus the results of an intervention should take such changes into account while interpreting results. According to Binzel, Field, and Pande [22] access to formal credit, such as availability of microfinance in an area, can similarly affect social networks, with formal credit access substituting for informal arrangements, reducing informal borrowing and gift exchange within the community.

A fascinating work most closely related to our study, is by Banerjee, Breza, Chandrasekhar, Duflo, Jackson, and Kinnan [23], where they focus on microfinance in particular and find social network shrinkage in two independent, empirical settings. In the first setting, a rural one, they find that communities which got access to microfinance experienced a shrinkage in social networks, even among individuals unlikely to borrow at all. They replicate these findings in a subsequent, urban setting in a randomised controlled trial. Microfinance exposure affected links even between those who were not borrowing from the microfinance institution, showing global spillovers. While the evidence offered by these studies is rigorous, experimental studies are sometimes criticised for their external validity [24–26].

The current study adds to the literature on social effects of financial interventions, by offering evidence on the relationship between microfinance and distrust. We use publicly available secondary datasets which precludes the possibility of measurement errors and/or any bias in data collection being correlated with outcomes of interest. This, and using cross-country data, makes the results relatively generaliseable. The hypothesis by itself, of a potential effect of microfinance on distrust in the low-income strata has not been previously studied to the best of our knowledge.

We follow the empirical approach of Kai and Hamori [27]; Imai, Gaiha, Thapa, and Annim [28]; and Hermes [29], among others, who study aggregate, country-level effects of microfinance [30–32]. Imai et al. [28] for instance, study the impact of microfinance on poverty levels across countries and find microfinance intensity to be associated with reduction in poverty, while Hermes [29] uses cross-country data to study the effect of microfinance on inequality, finding modest effects.

Our study follows the overall approach of these studies as we regress distrust levels on microfinance prevalence (henceforth, microfinance intensity, following the convention in literature) but we make the measurements more specific. For the dependent variable, distrust, we distinguish between distrust among the poor, the ultra-poor, and the rich. This helps us pin down the relationship for specific income strata. Similarly, for the explanatory variable, microfinance intensity in a country, we introduce a new measure, namely, the ratio of microfinance gross loan portfolio to the total domestic private credit in the country. We think that this measure of microfinance intensity represents an improvement on measures previously used in literature because of its higher precision as a measure of microfinance industry’s size in a country. Notwithstanding, we report results for two measures well-established in studies of aggregate effects of microfinance [28,29].

Lastly, a clarification before we proceed to action: we use the broader term ‘microfinance intensity’ to refer to what could more precisely be termed ‘microcredit intensity’, to situate the study seamlessly in the relevant literature, following earlier studies [28,29,31]. But the focus of the study, in particular, is on microfinance loans only i.e. microcredit.

## 2. Data & methods

### 2.1 Data

The study analyses the association between microfinance intensity and distrust levels among the poor. For cross-sectional regression, we use the latest available data of the year 2019 in WVS-EVS. Further, we use the empirical Bayes method on a 4-period panel for years 2001, 2007, 2012, and 2019, corresponding to the waves 4, 5, 6, and 7 respectively, of the World Values Survey. Table 1 provides variables’ description for quick reference while the summary statistics are reported in S1 Table.

**Table 1:** Variables.

Variable	Definition	Source
Distrust Poor	Distrust levels among the poor (Income levels 1, 2, 3 out of 10)	WVS
Distrust Ultra-Poor	Distrust levels among the ultra-poor (income level 1 out of 10)	WVS
Distrust Rich	Distrust level among the rich (income level 10 out of 10)	WVS
GLP/credit*	gross loan portfolio divided by total domestic private credit	MIX; WDI
GLP/capita*	gross loan portolio divided by total population	MIX; WDI
GLP/GDP*	(gross loan portfolio divided by GDP)*100	MIX; WDI
Fractionalisation	Ethnic fractionalisation index	Alesina et. al.; Drazanova
Top decile	Pre-tax share in national income of the top10% in the economy	WID
Yield*	Average real yield on gross loan portfolio 2011–2016 divided by its standard deviation	MIX

*These variables are log-transformed.

#### 2.1.1 Measuring microfinance intensity

Microfinance data comes from Microfinance Information Exchange (MIX), hosted by the World Bank Data Catalogue and the World Development Indicators. MIX Market data has been reported from 1999 to 2019, and covers the financial statements, outreach, and social performance data of microfinance institutions “targeting the unbanked” in developing economies [33]. MIX is targeted at achieving greater transparency in the microfinance industry, and while the data is self-reported, it follows predetermined formats, validation mechanisms, and standardisation for ease of comparison [28].

Gross loan portfolio (GLP)—the key component of our explanatory variable which is obtained from MIX—shows the total funds disbursed in loans by microfinance institutions (MFIs) and is adjusted for write-offs and inflation. We standardise this country-level GLP with respect to 3 alternative variables, yielding 3 different measures of microfinance intensity.

For the first measure we divide GLP by the total domestic credit to the private sector (GLP/credit), obtained from the World Development Indicators (WDI) [34]. To our knowledge, this is the first study to use the ratio of GLP to credit for measuring microfinance intensity. This yields a precise estimate of the salience of microfinance in the economy, as it normalises the GLP with respect to the size of the financial sector rather than a general country-wide measure. This gives us confidence that the results in this study are driven by microfinance, and not by overall financial development in the country.

For the second measure of microfinance intensity we divide GLP by the population of the country, which yields microfinance loans per capita (or simply GLP/capita) [28]. This measure is important in terms of indicating, potentially, the social quantum of microfinance in terms of its penetration within the life of the average person. The third measure is the same as the one used by Hermes [29] in which we take the GLP as a percentage of the GDP of a country. This indicates the significance of microfinance in relation to the size of a country’s economy.

#### 2.1.2 Measures of distrust

Distrust is the main outcome of interest in this study, the data for which is obtained from the WVS and EVS datasets [19]. WVS survey is conducted every 5 years, and EVS, every 9 years. For the latest, wave 7 of WVS, the WVS-EVS integrated data for 88 countries is available. The question from the survey that is used in the current study is, “Generally speaking, would you say that most people can be trusted or that you need to be very careful in dealing with people?”

The percentage of respondents answering “most people can be trusted” has been widely used as a measure of trust [35–37], and shown by Johnson and Mislin [38] to be positively correlated with experimentally measured trust. The proportion of people with the opposite response to the trust question i.e.“need to be very careful”, is used in this study as a measure of distrust, following the approach of Aghion, Algan, Cahuc, and Shleifer [39]. We focus on distrust as it is a relatively strongly felt emotion, and in the words of McKnight and Chervany [40] “fiery and frenzied” compared to “cool and collected” trust. Social frictions should appear more quickly and conspicuously in reported ‘distrust’ rather than ‘trust’.

The careful reader would note that the response “need to be very careful” indicates prudence and may simply be indicating reluctance to immediately trust people. Equating such ‘lack of trust’ with ‘distrust’ may be imprecise [41]. While this nuance may make a difference to questions that are directly affected by the difference between ‘lack of trust’ and ‘distrust’, for this paper, we think the point of the study remains irrespective of the label. We prefer the clearer and parsimonious ‘distrust’, but if the reader finds it more on-point the results can equally be interpreted using ‘lack of trust’.

Moreover, this study is concerned with ‘social distrust’, which is the absence of social/or generalised trust between strangers. This is related to, but different from, limited/particularised trust among knowns–friends and family—as well as civic/institutional trust, which represents trust in institutions, the governments, or the system [42,43]. While various forms of (dis)trust may follow similar paths, they are beyond the scope of the current work.

As the World Values Survey is conducted over a period of 5 to 6 years, we make a simplifying assumption for running meaningful regressions and for making comparison across waves possible: the distrust value for a given wave is assumed to represent the middle year of data collection. For the 4th Wave (1999–2004), this corresponds to the year 2001, for the 5th (2005–2009) to the year 2007, and so on. Notwithstanding, because distrust levels are unlikely to change substantially from year to year [44] the exact choice of year does not affect the overall results of the study.

Further, instead of dealing with a simple country-wide distrust measure, we distinguish distrust by income group. WVS asks respondents to rate their income on a 10-point scale, the lowest level being 1, and the highest level being “10”. We use this information to identify distrust in what we call the “ultra-poor”, corresponding to the lowest level in self-reported income, that is 1; the “poor”, corresponding to those on steps 1–3; and the “rich”, which captures those on the 10th step. This is important as exposure to microfinance is relevant to the poor and the ultra-poor only and should not have any effect on the rich.

#### 2.1.3 Control variables

Distrust in societies is correlated with ethnic heterogeneity as well as economic inequality, which we control for. All else equal, social distrust is positively related with ethnic heterogeneity as well as income inequality [45]. To account for ethnic heterogeneity, we use the ethnic fractionalisation index, which represents the probability that 2 people selected at random in a given population will be from different groups [46,47]. The higher the index value, the greater the probability. For economic inequality, we use the pre-tax income shares of the top 10% (top decile) of the population, following the approach of Piketty and Saez [48–50]. A higher value indicates higher inequality. We obtain this data from the World Inequality Database [51].

### 2.2 Methodology

The general linear regression model in panel takes the form,

$$Distrust_{it}=\alpha_{t}+\beta_{t}MF_{it-1}+\lambda_{t}Z_{it}+\varepsilon_{it}$$

Where, *MF* is a vector of 3 alternative measures of microfinance intensity; *Z* is a vector of covariates; *ε* is the random error term; i represents the country; and t represents the year.

Note that MF is modelled with a 1-year lag, capturing how microfinance intensity today is associated with distrust after a year. This is to be expected as the social effects of exposure to microfinance should take time to set in, especially to be noticeable enough to show in the data. Lag is common in literature on the determinants of trust [52,53].

#### 2.2.1 Cross-sectional regressions

For cross-sectional regression we use the ordinary least squares (OLS), the benchmark model for analysing association in continuous data in a single time period.

#### 2.2.2 Empirical Bayesian estimation

For analysing panel, we use empirical Bayes method. Empirical Bayes was pioneered by Efron & Morrison [54–57] and lies at the intersection of the classical Bayesian and the frequentist approach to statistics. It is frequentist in the sense that it tries to get at the underlying parameters, but Bayesian in the sense that it draws on the very data to recreate a prior distribution, from which a posterior distribution is reached. The bias and imprecision that a small sample suffers from, the empirical Bayes method helps mitigate by generating a prior distribution from given data, from which hyperparameters are estimated to get at a posterior distribution yielding more stable results with lower standard errors, less susceptible to bias arising from erratic outliers [57–61]. This gives us confidence that the relationship between microfinance intensity and distrust is not driven by extremely strong association in any particular year as such extreme co-variations are allocated lower weights in proportion to their deviation from the universe of values.

We follow the general Bayesian panel approach taken by Carrington and Zaman [58] of the form,

$$Y_{it}=\beta_{i}X_{it}+\varepsilon_{it}$$ (i)

∀i=1,2,…,N

t=1,2…,T

Where *Y* represents the dependent variable; *X* represents the independent variable and the covariates; *t* represents the number of time periods; *i* denotes the number of countries; *β* is the coefficient vector; *N* represents the total no. of countries; *T* represents the total time-periods; and *ε* is the random error component.

In matrix form,

$$Y_{i}={\left[\begin{array}{c} y_{i1}\\ y_{i2}\\ .\\ .\\ .\\ y_{iT} \end{array}\right]}_{T\times 1}X_{i}={\left[\begin{array}{c} x_{i1}\\ x_{i2}\\ .\\ .\\ .\\ x_{iT} \end{array}\right]}_{T\times K}\varepsilon_{i}={\left[\begin{array}{c} \varepsilon_{i1}\\ \varepsilon_{i2}\\ .\\ .\\ .\\ \varepsilon_{iT} \end{array}\right]}_{T\times 1}$$

$${\mathrm{Where},\;X}_{it}=[\begin{array}{cccc} X_{it}^{1} & X_{it}^{2} & \ldots & X_{it}^{k} \end{array}]k‐\mathrm{regressors}$$

$$\mathrm{and},\;\varepsilon_{i}∼N(0,\delta_{i}^{2})$$

The data density is,

βi^β∼N(β,Ωi) (ii)

It is important to note here that this data density is as such, in contrast with the prior density, which is based on previous knowledge or belief about the data. The prior density, may be given by,

β∼N(μ,Λ) (iii)

So, the posterior density becomes [60],

ββi^:N(mi,Vi) (iv)

Where *m*_*i*_ and *V*_*i*_ are,

$$m_{i}=V_{i}({{\hat{\Omega }}_{i}}^{-1}\hat{\beta i}+\Lambda^{-1}\mu )\;and\;V_{i}={\left({{\hat{\Omega }}_{i}}^{-1}+\Lambda^{-1}\right)}^{-1}$$ (v)

As the Classical Bayes estimator is the mean of posterior, Eq (v) may be written as,

$${\hat{\beta i}}_{\left(CB\right)}=V_{i}({{\hat{\Omega }}_{i}}^{-1}\hat{\beta i}+\Lambda^{-1}\mu )$$

and its variance covariance matrix is,

$$V_{i}={\left({{\hat{\Omega }}_{i}}^{-1}+\Lambda^{-1}\right)}^{-1}$$

But the two hyperparameters i.e. *μ* and Λ are unknown. The empirical Bayes method uses the available data to estimate these hyperparameters and construct prior distribution from the data itself instead of previous knowledge or belief. Thus, the empirical Bayes estimator may be written as,

$${\hat{\beta i}}_{\left(EB\right)}=\hat{V_{i}}({{\hat{\Omega }}_{i}}^{-1}\hat{\beta i}+{\hat{\Lambda }}^{-1}\hat{\mu })$$ (vi)

Where the estimated hyperparameters are,

$$\hat{\Lambda }={\left(\sum_{i=1}^{N}{{\hat{\Omega }}_{i}}^{-1}\right)}^{-1}$$

and,

$$\hat{\mu }=\hat{\Lambda }\left(\sum_{i=1}^{N}{{\hat{\Omega }}_{i}}^{-1}\hat{\beta i}\right)$$

#### 2.2.3 Two-stage least squares

While the empirical Bayesian analysis minimises the problem posed by extreme values, it does not address the issue of potential endogeneity. Microfinance intensity and distrust among the poor may be correlated with a third omitted variable, for instance, which affects both. Similarly, microfinance intensity may be higher in environments with higher levels of distrust. The association observed could be affected by any such unobserved relationships.

The 2-Stage Least Squares (2SLS) allows us to account for these concerns, but a novel instrument is needed which is associated with microfinance intensity 2018 but not distrust. We use the real inflation-adjusted yield on the gross loan portfolio, which measures the interest revenues of microfinance institutions, as an instrument. Specifically, because an MFI’s decision to expand the loan portfolio in a particular year is affected by past interest earnings—and their stability—[62,63] we divide the average real yield of gross loans over the 2011–2016 period by the standard deviation of this yield over the same period. We see no reason to expect distrust to be correlated with the past microfinance yield in a country. Any association of distrust and the standard deviation of the yields is even less plausible. Thus, yield, adjusted for its volatility, makes for a good instrument correlated with microfinance intensity but not distrust.

## 3. Results

### 3.1 Cross-sectional regressions

Table 2 shows the estimates from cross-section OLS regressions for the year 2019, where the main columns indicate distrust level by income group and sub-columns (1), (2), and (3) denote independent regressions using GLP/credit, GLP/capita, and GLP/GDP as the explanatory variable, respectively. Microfinance intensity is positively associated with the distrust levels among the poor at 1% level. The higher the intensity of microfinance in an economy, the higher are distrust levels among the poor, which suggests an effect of microfinance on relatively poor communities that are generally the target population of MFIs.

**Table 2:** Results of 2019 OLS (dependent variable: Distrust—by income group).

	Distrust Poor			Distrust Ultra-poor			Distrust Rich		
	(1)	(2)	(3)	(1)	(2)	(3)	(1)	(2)	(3)
GLP/credit	0.033***			0.033**			0.042		
	[0.011]			[0.013]			[.025]		
GLP/capita		0.032***			0.028			0.032	
		[0.013]			[.018]			[.026]	
GLP/GDP			0.031***			0.028			0.028
			[.011]			[.0172]			[.025]
Fractionalisation	0.119*	0.117**	0.107**	0.098	0.161*	0.152*	0.192	0.142	0.134
	[0.063]	[0.054]	[0.054]	[0.075]	[.088]	[0.089]	[.141]	[.126]	[.128]
Top Decile	0.625***	0.609***	0.633***	0.601**	0.188	0.207	0.572	0.496	0.530
	[.219]	[0.208]	[.204]	[0.258]	[.337]	[.334]	[.487]	[.483]	[.481]
Constant	0.575***	0.481***	0.526***	0.612***	.662***	0.703***	0.551**	0.501**	0.539**
	[0.107]	[0.094]	[0.096]	[0.127]	[0.152]	[.156]	[.239]	[.218]	[.225]
F-statistic	9.43***	9.48***	9.48***	6.05***	2.42*	2.53*	2.84*	1.680	1.590
P > F	0.000	0.000	0.000	0.002	0.082	0.073	0.054	0.189	0.209

Standard errors in brackets.

*p < 0.1

**p < 0.05

***p < 0.01.

The effects of microfinance intensity on the ultra-poor are significant only with the first measure of intensity, and at the 5% level. This indicates a weaker relationship of microfinance intensity with the ultra-poor which could be due to the ultra-poor being ‘left behind’ even by financial inclusion initiatives [64]. Because of this they may be less exposed to the immediate effects on trust levels, though still exposed to spillover effects [23] from other people’s exposure, and a generalised rise in distrust in neighbourhoods. Together, the results suggest an effect of microfinance on the low-income population, generally i.e. the target population of MFIs. These aggregate effects may be read in line with earlier studies, which point to the negative externalities in social network effects of microfinance that go beyond the immediate borrowers [23]. If microfinance has effect on distrust levels, it should impact only low-income people, not affecting the rich. Indeed, we don’t find a statistically significant relationship between microfinance and distrust in regressions of any of the three measures of microfinance intensity. As expected, both control variables, fractionalisation and top decile income, are positively associated with distrust.

### 3.2 Empirical Bayes

Tables 3–5 report the estimates for empirical Bayes panel with GLP/credit, GLP/capita, and GLP/GDP respectively as the main explanatory variable. For each income group, the first column indicates empirical Bayesian priors (henceforth, Bayesian prior). Bayesian prior is a precision-weighted average of OLS estimates for 2001, 2007, 2012, and 2019. Estimates from the Bayesian prior may be conceptualised in how they differ from the pooled OLS: Bayesian prior assumes heteroscedasticity across different cross-sections in contrast to the pooled OLS which assumes homoscedasticity. In the WVS data, it is likely that the variation of distrust across different time periods will not be the same. This may be due to a number of factors. For example, the variance of the incremental sample for every new wave may be correlated with some ‘type’ of countries added, which may affect overall variance. Another potential channel affecting variance across cross-sections may be shocks, such as Covid [65–70] in Wave 7, in which a tighter distribution of distrust is likely. The priors show microfinance intensity to be associated with distrust in poor as well as the ultra-poor according to all 3 measures of microfinance intensity.

**Table 3:** Empirical Bayes (dependent variable: Distrust by income group; explanatory variable: GLP/credit).

	Distrust Poor					Distrust Ultra-poor						Distrust Rich			
	Priors	2019	2012	2007	2001	Priors	2019	2012	2007	2001	Priors	2019	2012	2007	2001
GLP/credit	0.042***	0.040***	0.042***	0.040***	0.046***	0.027***	0.029***	0.028***	0.025**	0.026***	-0.008	0.006	-0.012	-0.024	-0.004
	[0.008]	[.007]	[.008]	[.008]	[.008]	[0.010]	[0.008]	[0.009]	[0.009]	[0.009]	[0.016]	[0.013]	[0.014]	[0.014]	[0.015]
Fractionalisation	0.108**	0.112***	0.112***	0.102**	0.108**	0.093*	0.093**	0.089*	0.086*	0.105**	-0.002	0.042	-0.051	0.022	-0.016
	[0.044]	[.025]	[.040]	[.042]	[.039]	[0.052]	[.043]	[0.048]	[0.049]	[0.048]	[0.083]	[0.072]	[0.072]	[0.076]	[0.080]
Top decile	0.396	0.455***	0.397***	0.365**	0.362**	0.277	0.376**	0.264	0.198	0.251	0.274	0.348	0.390	0.089	0.270
	[.150]	[.123]	[.136]	[.139]	[.139]	[0.173]	[0.143]	[0.156]	[0.158]	[0.164]	[0.271]	[0.235]	[0.233]	[0.240]	[0.269]
Constant	0.690	0.657***	0.684***	0.693***	0.707***	0.748***	0.708***	0.759***	0.779***	0.753***	0.658***	0.641***	0.621***	0.692***	0.671***
	[.074]	[.060]	[.004]	[.070]	[.070]	[.086]	[0.070]	[0.077]	[0.079]	[0.082]	[0.135]	[0.117]	[0.114]	[0.122]	[0.133]

Standard errors in brackets.

*p < 0.1

**p < 0.05

***p < 0.01.

**Table 4:** Empirical Bayes (dependent variable: Distrust by income group; explanatory variable: GLP/capita).

	Distrust Poor					Distrust Ultra-poor					Distrust Rich				
	Priors	2019	2012	2007	2001	Priors	2019	2012	2007	2001	Priors	2019	2012	2007	2001
GLP/capita	0.041***	0.039***	0.043***	0.042***	0.043***	0.030***	.030***	0.033***	0.03***	0.028***	0.016	0.021	0.015	0.007	0.019
	[0.008]	[0.007]	[0.008]	[0.008]	[0.008]	[0.011]	[0.009]	[0.010]	[0.070]	[0.010]	[0.016]	[0.014]	[0.015]	[0.014]	[0.015]
Fractionalisation	.0945**	0.103***	.010***	0.082**	0.086**	0.087	0.106**	0.082*	0.066	0.089	-0.049	0.005	-0.096	-0.049	-0.058
	[0.041]	[0.033]	[0.038]	[0.040]	[0.039]	[0.054]	[0.046]	[0.048]	[0.200]	[1.806]	[0.079]	[0.067]	[0.069]	[0.073]	[0.074]
Top decile	0.444***	0.487***	0.428***	0.420***	0.438***	0.077	0.099	0.091	0.037	0.086	0.410	0.425	0.526	0.250	0.432
	[0.144]	[0.118]	[0.130]	[0.134]	[0.135]	[0.181]	[0.159]	[0.161]	[0.163]	[0.610]	[0.271]	[0.236]	[0.233]	[0.245]	[0.261]
Constant	0.536***	0.522***	0.537***	0.547***	0.539***	0.748***	0.729	.744***	0.773	0.745	0.620***	0.591***	0.589***	0.697***	0.606***
	[0.066]	[0.054]	[0.060]	[0.061]	[0.062]	[0.083]	[0.073]	[0.000]	[0.000]	[0.000]	[0.126]	[0.109]	[0.110]	[0.114]	[0.121]

Standard errors in brackets.

*p < 0.1

**p < 0.05

***p < 0.01.

**Table 5:** Empirical Bayes (dependent variable: Distrust by income group; explanatory variable: GLP/GDP).

	Distrust Poor					Distrust Ultra-poor						Distrust Rich			
	Priors	2019	2012	2007	2001	Priors	2019	2012	2007	2001	Priors	2019	2012	2007	2001
GLP/GDP	0.044***	0.039***	0.045***	0.048***	0.046***	0.036***	0.033***	0.034***	0.038***	0.040***	0.020	0.023	0.015	0.019	0.020
	[0.008]	[0.033]	[0.008]	[0.008]	[0.008]	[0.011]	[0.010]	[0.010]	[0.011]	[0.011]	[0.018]	[0.014]	[0.015]	[0.017]	[0.017]
Fractionalisation	0.082*	0.092***	0.081**	0.692*	0.080**	0.081	0.102**	0.066	0.065	0.090*	-0.014	0.029	-0.068	-0.009	-0.012
	[0.041]	[0.033]	[0.038]	[0.039]	[0.038]	[.055]	[0.047]	[0.050]	[0.052]	[0.050]	[0.081]	[0.069]	[0.072]	[0.076]	[0.075]
Top decile	0.383**	0.456***	0.395***	0.353**	0.316	0.095	0.121	0.137	0.075	0.046	0.517	0.497**	0.622**	0.432	0.527*
	[0.0146]	[0.119]	[0.135]	[0.138]	[0.136]	[0.194]	[0.167]	[0.174]	[0.180]	[0.175]	[0.298]	[0.252]	[0.262]	[0.278]	[0.279]
Constant	0.630***	0.599***	0.627***	0.646***	0.653***	0.782***	0.761***	0.776***	0.797***	0.795***	0.567***	0.571***	0.540***	0.600***	0.548***
	[0.071]	[0.057]	[0.065]	[0.068]	[0.066]	[0.096]	[0.081]	[0.085]	[0.090]	[0.087]	[0.147]	[0.122]	[0.129]	[0.138]	[0.138]

Standard errors in brackets.

*p < 0.1

**p < 0.05

***p < 0.01.

The priors as well as the year-wise posterior estimates are statistically significant at the 1% level for the poor as well as the ultra-poor for all 3 measures of microfinance intensity. This is in sharp contrast to distrust among the rich, which shows no significant association with any measure of microfinance. Furthermore, in contrast to the OLS estimates, distrust in the ultra-poor is also significant in empirical Bayes results. This may be due to greater precision achieved in panel due to more data and the use of empirical Bayes. In other words, while the effects of microfinance intensity on distrust among the ultra-poor are smaller than the poor, empirical Bayes estimates are able to capture these effects. This may be seen in relatively smaller coefficients of microfinance intensity in Tables 4–6, for distrust in the ultra-poor compared to those for distrust among the poor, as well as slightly weak significance (but still having p<0.01). As discussed earlier, this is to be expected as microfinance loans often fail to reach the ultra-poor [71]. Lastly, it is important to note that empirical Bayes estimates have lower standard errors for the estimated coefficient of microfinance intensity for almost all specifications reflecting the gain in precision achieved from using empirical Bayes.

**Table 6:** Results of 2nd Stage of 2019 2SLS (dependent variable: Distrust by income group).

	Distrust Poor			Distrust Ultra-poor			Distrust Rich		
	(1)	(2)	(3)	(1)	(2)	(3)	(1)	(2)	(3)
GLP/credit	0.101**			0.091*			0.107		
	[0.049]			[0.053]			[0.084]		
GLP/capita		0.078**			0.092*			0.073	
		[0.031]			[0.049]			[0.064]	
GLP/GDP			0.083**			0.099*			0.079
			[0.035]			[0.056]			[0.070]
Fractionalisation	0.092	0.142**	0.106	0.077	0.201*	0.158	0.182	0.167	0.133
	[0.096]	[0.064]	[0.069]	[0.103]	[0.103]	[0.110]	[0.163]	[0.132]	[0.138]
Top decile	0.618*	0.480*	0.567**	0.544	-0.712	-0.020	0.793	0.545	0.627
	[0.318]	[0.261]	[.269]	[0.341]	[0.419]	[0.428]	[0.540]	[0.539]	[0.540]
Constant	0.724***	0.475***	0.581***	0.761***	0.712***	0.838***	0.568*	0.410*	0.510**
	[0.183]	[0.115]	[0.128]	[0.196]	[0.185]	[0.204]	[0.311]	[0.238]	[0.257]
F stat (first stage)	4.022	9.741	7.090	4.022	9.741	7.090	4.022	9.741	7.090
Prob > F (first stage)	0.055	0.004	0.012	0.055	0.004	0.012	0.055	0.004	0.012

Standard errors in brackets.

*p < 0.1

**p < 0.05

***p < 0.01

### 3.3 Two-squared least squares (2SLS)

In the first stage, all 3 measures of microfinance intensity show a statistically significant relationship with the instrumental variable (IV) yield (S2 Table). In the second stage estimates (Table 6), GLP/credit, GLP/capita, GLP/GDP show a statistically significant association with distrust in the poor as well as the ultra-poor. Distrust in the rich remains insignificant, as in earlier regressions. Also, again in 2SLS, the results for the ultra-poor are slightly weaker, than relatively larger category of poor.

It may be noted that second-stage estimates are subject to the strength of the instrument, indicated in F-statistic. The F-statistic of 9.7 for GLP/capita almost meets the rule of thumb value of 10, suggested by Staiger, Stock, and Watson [72] for unbiased estimates. The value for GLP/credit and GLP/GDP, although significantly related to the instrumental variable (S2 Table), falls short of meeting the F-statistic of 10.

To ensure that the results are not an artefact of a potentially weak instrument, we estimate weak instruments-robust [73,74] Conditional Likelihood Ratio, Lagrange Multiplier Score, and Anderson-Rubin Statistic, and report coefficient, p-values, and coverage-corrected confidence sets, as suggested by Andrews, Stock, and Sun [75]. The results reported in Tables 7 and 8, show all 3 measures of microfinance to have a significant effect on distrust among the poor and the ultra-poor respectively.

**Table 7:** Weak instrument-robust 2SLS (dependent: Distrust poor).

LIML estimate of beta (GLP/credit) = 0.100798			
Test	Confidence Set		P-value
Conditional LR	[0.0288419,	23.62876]	0.0087
Anderson-Rubin	[0.0288419,	23.62832]	0.0087
Score (LM)	[0.0288419,	23.62832]	0.0087
LIML estimate of beta (GLP/capita) = 0.077772			
Test	Confidence Set		P-value
Conditional LR	[0.0251332	.214914]	0.0043
Anderson-Rubin	[0.0251332	.214914]	0.0043
Score (LM)	[0.0251332	.214914]	0.0043
LIML estimate of beta (GLP/GDP) = 0.0833191			
Test	Confidence Set		P-value
Conditional LR	[0.0272524,	0.3064244]	0.0087
Anderson-Rubin	[0.0272524,	0.3064244]	0.0087
Score (LM)	[0.0272524,	0.3064244]	0.0087

**Table 8:** Weak instrument-robust 2SLS (dependent: Distrust ultra-poor).

LIML estimate of beta (GLP/credit) = 0.0911413			
Test	Confidence Set		P-value
Conditional LR	[0.0072805,	23.42728]	0.0350
Anderson-Rubin	[0.0072805,	23.42753]	0.0087
Score (LM)	[0.0072805,	23.42753]	0.0087
LIML estimate of beta (GLP/capita) = 0.0920796			
Test	Confidence Set		P-value
Conditional LR	[0.0075603	0.3124471]	0.0320
Anderson-Rubin	[0.0075603	0.3124471]	0.0320
Score (LM)	[0.0075603	0.3124471]	0.0320
LIML estimate of beta (GLP/GDP) = 0.0833191			
Test	Confidence Set		P-value
Conditional LR	[0.0075603,	0.3124471]	0.0320
Anderson-Rubin	[0.0075603,	0.3124471]	0.0320
Score (LM)	[0.0075603,	0.3124471]	0.0320

To summarise, the findings from OLS, empirical Bayes, as well as instrument variable estimation show microfinance to be associated with a worsening of trust levels, in line with earlier studies which find a negative effect of microfinance on various constituents of social capital including trust [18,76,77].

## 4. Discussion and concluding remarks

While the social effects of microfinance are extensively documented in qualitative studies, there are still few empirical studies on the subject. This paper contributes to this literature, studying the association between microfinance intensity and social distrust. The results, based on OLS and empirical Bayes, show an association between microfinance intensity and distrust among the low-income strata in a country. Moreover, using instrumental variable for microfinance intensity, we find evidence in favour of the hypothesis that microfinance affects distrust. We don’t find any association between microfinance and distrust levels among the rich in our sample, potentially because the rich are not exposed to microfinance.

The findings of this study are in line with earlier studies, which report economic interventions can have social consequences. In particular the results are closely related to findings of Banerjee, Breza, Chandrasekhar, Duflo, Jackson, and Kinnan [23] who report overall weakening of social networks in communities upon greater exposure to microfinance. According to the analysis presented in this paper, one explanation for social network shrinkage found in their [23] study may be higher levels of distrust in people. In fact, the relationship between microfinance intensity and distrust in the low-income strata found in our study shows in country-level data, perhaps because it has effects on not only borrower-to-borrower social links, but also on borrower to non-borrower, and non-borrower to non-borrower links [23]. This suggests general equilibrium effects within the low-income communities in countries with high prevalence of microfinance.

But why does microfinance have social effects? One possible mechanism in presented in earlier research [23] is that the availability of microfinance in an area reduces the dependence on informal credit institutions within a community which people previously rely on, acting as a substitute to pre-existing informal credit. Thus, people socialise less because they are less financially dependent on each other. Therefore, social networks can shrink [23]. In the same vein, it may be argued that if trusting behaviour is an outcome of socialisation, rather than vice versa, distrust can arise from comparatively less socialisation in communities with higher microfinance exposure. In short, formal credit options reduce the need to socialise, people interact less resultantly, and this affects their ability or inclination to trust strangers over time. The relationship between social interaction and social trust is already established in literature [78,79].

While we do not rule this out, we propose another channel by which microfinance may lead to distrust: the use of social as opposed to physical collateral. Traditionally, microfinance institutions have used some variation of the group-lending model in which peers come together to form a borrowing group. While defaults/delinquencies may not show up on the balance sheets of microfinance institutions as other members are obliged to pay on behalf of the defaulting member owing to joint liability [80,81], such instances may be common within groups. This can happen explicitly, for example, when the group has to dig into the collective pre-emptive savings account being maintained alongside the loan account to compensate for a member’s default (note that even this default doesn’t appear in MFI’s balance sheets) [82], or implicitly when another member pays an instalment on someone’s behalf, in expectation of it being a temporary arrangement. The *incidence* of repayment, thus, may not be equal across members across time [83]. Worse, a group-member who is not eyeing future loans may–or may be deemed to have–default(ed) strategically. Whether explicit or implicit, a delinquency or default, under genuine financial distress or strategic, such incidences result in peer pressure, social sanctions and/or social exclusion, sometimes with the backing of MFI staff, trying to recover a loan [84]. Such problems can fracture social ties, leading to distrust [85].

With the sheer rise in outreach of microfinance, and the strict discipline borrowers are required to show, sometimes with a repayment schedule demanding even weekly payments, such incidences are all the more likely. Most of the borrowers, as may be expected of any random sample of human population, are not ‘natural’ entrepreneurs, and their incomes are unlikely to grow significantly—even less, as fast as the interest rates in the commercially oriented micro-loans project. They are liquidity-constrained–which tempts them into borrowing—but financially unsophisticated–which makes repayment difficult. Thus, delinquencies and default are common, even if they don’t appear in MFI’s books and can have social consequences.

More recently, there has been a rise in individual, alongside group-lending in microfinance. But the mechanisms these models follow for unsecured credit again rely on social capital of the borrower, where a guarantor partially substitutes for physical collateral. This, again, leverages social capital, specifically trust, sometimes possibly beyond what may be socially desirable.

From a regulator’s point of view, it is important to cap interest rates within reasonable limits and to promote responsible lending and recovery practices. For their part, microfinance institutions need to be conservative, rather than liberal in lending (even though most literature pushes the latter) to decrease the rate of default and its social repercussions. Meanwhile, scholars of joint liability need to solve the paradox of social sanctions having to drive exclusion rather than inclusion if the threat of punishment is at all to be credible and group-loans are to work. If the cost of financial inclusion is social exclusion, in other words, we need to know what the numbers are, and when and where may the trade-off be worth it.

In this paper we, (i) introduced GLP/credit as a measure of microfinance intensity; (ii) used volatility-adjusted past portfolio yield as an instrument for microfinance intensity; and (iii) investigated the effect of microfinance on distrust across countries using 3 different measures of microfinance intensity, using distrust data for 3 income groups, employing multiple estimation techniques. This is the first study to show that microfinance is associated with a worsening of generalised trust levels using empirical data.Yet, the findings presented here can benefit from more data especially using alternative measures of trust and utilising other datasets. Moreover, the relationship of microfinance loans with distrust should ideally be corroborated with micro-level evidence. Future research can also explore the effects of microfinance on other social and psychological indicators of wellbeing.

While there is a thick strand of theoretic and empirical literature on incentive structures in microfinance from the lender’s point of view, there is a need to understand the social mechanisms which make these incentive structures work for the lender–perhaps, the incentive structure beneath the incentive structure. Do unethical coercive practices for recovery, forced liquidation of assets, deterioration of relationships, and extreme distress–as reported in sociology studies [76,77,86]–lie behind the decent repayment rates, low default risk, and the magic of joint liability that we see in many empirical studies? Research at the intersection of economics and sociology can help break down these issues into workable hypotheses providing much needed insight into what works for the debtor as well as the creditor.

## Supporting information

S1 Table Summary statistics. (DOCX)

S2 Table First stage of 2SLS. (DOCX)

S1 Data (XLSX)
