# Supplementary material for: Till debt does us apart: Cross-country evidence on the relationship between microfinance prevalence and social distrust
Source: PLoS One. 2023 Mar 8;18(3):e0282072. doi: 10.1371/journal.pone.0282072 (PMC9994719; doi:10.1371/journal.pone.0282072)
Supplement: S1 Table — (DOCX) [file pone.0282072.s001.docx]

**S1. Table. Summary Statistics**

|  |  |  |  |  |  |  |  |  |  |  |
| --- | --- | --- | --- | --- | --- | --- | --- | --- | --- | --- |
|  | **S1. Table. *Summary Statistics*** | | | | | | | | |  |
|  | **Year** | **Variable** | **No.** | **Mean** | **St. Dev.** | **Min** | **Max** | **Skewness** | **Kurtosis** |  |
|  | **2019** | Distrust Poor | 50 | 0.831 | 0.102 | 0.408 | 0.975 | -1.756 | 7.242 |  |
|  |  | Distrust Ultra-Poor | 50 | 0.85 | 0.13 | 0.333 | 1 | -1.93 | 7.369 |  |
|  |  | Distrust Rich | 50 | 0.803 | 0.186 | 0.068 | 1 | -1.691 | 6.864 |  |
|  |  | GLP to private credit ratio | 36 | 0.058 | 0.11 | 0 | 0.458 | 2.691 | 7.147 |  |
|  |  | GLP to population ratio | 40 | 83.154 | 191.96 | 0.002 | 1020.42 | 3.689 | 15.121 |  |
|  |  | GLP as percentage GDP | 40 | 2.066 | 5.002 | 0 | 28.756 | 4.21 | 21.937 |  |
|  |  | Ethnic Fractionalisation Index* | 49 | 0.408 | 0.239 | 0.025 | 0.855 | 0.072 | 2.049 |  |
|  |  | Income share of richest 10% | 50 | 0.438 | 0.074 | 0.269 | 0.589 | -0.055 | 2.673 |  |
|  | **2012** | Distrust Poor | 30 | 0.803 | 0.137 | 0.357 | 0.97 | -1.23 | 4.79 |  |
|  |  | Distrust Ultra-Poor | 30 | 0.859 | 0.134 | 0.454 | 1 | -1.595 | 5.054 |  |
|  |  | Distrust Rich | 30 | 0.807 | 0.182 | 0.418 | 1 | -0.574 | 2.07 |  |
|  |  | GLP to private credit ratio | 41 | 0.077 | 0.126 | 0 | 0.52 | 2.192 | 7.277 |  |
|  |  | GLP to population ratio | 45 | 88.548 | 135.391 | 0.022 | 597.659 | 2.207 | 7.734 |  |
|  |  | GLP as percentage GDP | 45 | 2.328 | 3.356 | 0 | 13.959 | 1.873 | 6.168 |  |
|  |  | Ethnic Fractionalisation Index | 48 | 0.41 | 0.24 | 0.026 | 0.855 | 0.032 | 2.03 |  |
|  |  | Income share of richest 10% | 50 | 0.441 | 0.079 | 0.296 | 0.617 | 0.116 | 2.386 |  |
|  | **2007** | Distrust Poor | 26 | 0.748 | 0.146 | 0.456 | 0.947 | -0.635 | 2.343 |  |
|  |  | Distrust Ultra-Poor | 25 | 0.812 | 0.13 | 0.56 | 0.976 | -0.616 | 1.937 |  |
|  |  | Distrust Rich | 21 | 0.769 | 0.205 | 0.302 | 1 | -0.49 | 2.371 |  |
|  |  | GLP to private credit ratio | 34 | 0.057 | 0.1 | 0 | 0.483 | 2.734 | 11.098 |  |
|  |  | GLP to population ratio | 45 | 30.296 | 40.024 | 0.004 | 174.274 | 1.715 | 5.859 |  |
|  |  | GLP as percentage GDP | 45 | 1.491 | 2.022 | 0 | 7.697 | 1.672 | 5.06 |  |
|  |  | Ethnic Fractionalisation Index | 48 | 0.413 | 0.242 | 0.028 | 0.856 | 0.006 | 2.016 |  |
|  |  | Income share of richest 10% | 50 | 0.451 | 0.078 | 0.306 | 0.593 | -0.25 | 2.094 |  |
|  | **2001** | Distrust Poor | 22 | 0.731 | 0.119 | 0.494 | 0.906 | -0.46 | 2.276 |  |
|  |  | Distrust Ultra-Poor | 22 | 0.785 | 0.142 | 0.44 | 1 | -1.045 | 3.847 |  |
|  |  | Distrust Rich | 20 | 0.662 | 0.183 | 0.312 | 1 | -0.541 | 2.616 |  |
|  |  | GLP to private credit ratio | 17 | 0.002 | 0.006 | 0 | 0.025 | 3.21 | 12.053 |  |
|  |  | GLP to population ratio | 30 | 1.928 | 4.753 | 0.001 | 25.626 | 4.336 | 22.068 |  |
|  |  | GLP as percentage GDP | 27 | 0.167 | 0.479 | 0 | 2.569 | 4.433 | 22.627 |  |
|  |  | Ethnic Fractionalisation Index | 49 | 0.413 | 0.241 | 0.024 | 0.857 | 0.009 | 2.001 |  |
|  |  | Income share of richest 10% | 50 | 0.452 | 0.09 | 0.293 | 0.63 | 0.001 | 1.961 |  |
|  | Note: GLP data from t-1 e.g. for 2019, GLP measures used are from 2018. GLP statistics are before taking log. | | | | | | | | |  |
|  | *Value of 2013, the latest available figure. | | | | | | | | |  |
