# Supplementary material for: Till debt does us apart: Cross-country evidence on the relationship between microfinance prevalence and social distrust
Source: PLoS One. 2023 Mar 8;18(3):e0282072. doi: 10.1371/journal.pone.0282072 (PMC9994719; doi:10.1371/journal.pone.0282072)
Supplement: S2 Table — (DOCX) [file pone.0282072.s002.docx]

**S2. Table. First Stage of 2SLS**

| **S2. Table. First Stage of 2SLS** | | | |  |
| --- | --- | --- | --- | --- |
|  |  |  |  |  |
|  | GLP/credit | GLP/capita | GLP/GDP |  |
| Yield | 1.127* | 1.442*** | 1.346** |  |
|  | [0.562] | [0.462] | [0.506] |  |
| Fractionalisation | 0.911 | -0.148 | 0.574 |  |
|  | [0.883] | [0.668] | [0.731] |  |
| Top decile | -0.861 | 1.506 | 0.360 |  |
|  | [3.232] | [2.633] | [2.881] |  |
| Constant | -3.017 | -0.854 | -2.074 |  |
|  | [1.493] | [1.239] | [1.356] |  |
| No. of observations | 30 | 34 | 34 |  |
| F statistic | 4.023 | 9.741 | 7.090 |  |
| Standard errors in brackets. *p < 0.1; **p < 0.05; ***p < 0.01 | | | |  |
